# Deficiência de Ferro na Insuficiência Cardíaca com Fração de Ejeção Reduzida: Fisiopatologia, Diagnóstico e Tratamento

**DOI:** 10.36660/abc.20201257

**Published:** 2022-03-10

**Authors:** Guilherme Augusto Reissig Pereira, Luís Beck-da-Silva

**Affiliations:** 1 Hospital São Lucas Pontifícia Universidade Católica do Rio Grande do Sul Porto Alegre RS Brasil Hospital São Lucas da Pontifícia Universidade Católica do Rio Grande do Sul, Porto Alegre, RS – Brasil; 2 Programa de Pós-Graduação em Cardiologia Universidade Federal do Rio Grande do Sul Porto Alegre RS Brasil Programa de Pós-Graduação em Cardiologia – Universidade Federal do Rio Grande do Sul, Porto Alegre, RS – Brasil; 3 Hospital de Clínicas de Porto Alegre Porto Alegre RS Brasil Hospital de Clínicas de Porto Alegre, Porto Alegre, RS – Brasil

**Keywords:** Ferro, Deficiência de Ferro, Insuficiência Cardíaca Sistólica

## Abstract

A deficiência de ferro (DF) ou ferropenia é uma importante comorbidade na insuficiência cardíaca com fração de ejeção reduzida (ICFER) estável, e muito prevalente tanto nos anêmicos como não anêmicos. A ferropenia na ICFER deve ser pesquisada por meio da coleta de saturação de transferrina e ferritina. Há dois tipos de ferropenia na IC: absoluta, em que as reservas de ferro estão depletadas; e funcional, onde o suprimento de ferro é inadequado apesar das reservas normais. A ferropenia está associada com pior classe funcional e maior risco de morte em pacientes com ICFER, e evidências científicas apontam melhora de sintomas e de qualidade de vida desses pacientes com tratamento com ferro parenteral na forma de carboximaltose férrica. O ferro exerce funções imprescindíveis como o transporte (hemoglobina) e armazenamento (mioglobina) de oxigênio, além de ser fundamental para o funcionamento das mitocôndrias, constituídas de proteínas à base de ferro, e local de geração de energia na cadeia respiratória pelo metabolismo oxidativo. A geração insuficiente e utilização anormal de ferro nas células musculares esquelética e cardíaca contribuem para a fisiopatologia da IC. A presente revisão tem o objetivo de aprofundar o conhecimento a respeito da fisiopatologia da ferropenia na ICFER, abordar as ferramentas disponíveis para o diagnóstico e discutir sobre a evidência científica existente de reposição de ferro.

## O Problema Clínico

A insuficiência cardíaca (IC) tem sido considerada um problema de saúde pública global que afeta 26 milhões de pacientes no mundo todo.^1^ O número de pacientes com IC em 2015 no Brasil foi de aproximadamente 2.846.000, correspondendo à 2% da população adulta, sendo que ocorre aumento da prevalência com o avançar da idade.^2^

Em registro brasileiro de pacientes internados por IC de diferentes regiões do Brasil, a mortalidade intra-hospitalar encontrada foi de 12,6%.^3^ Além da alta mortalidade intra-hospitalar, estima-se que 50% dos pacientes diagnosticados com IC estarão mortos dentro de um período de 5 anos.^4 , 5^ Outro ponto que merece destaque é o impacto financeiro da doença. Em 2015 a IC gerou no Brasil um custo substancial de R$ 22,1 bilhões de reais.^2^

A anemia é uma doença comum na insuficiência cardíaca com fração de ejeção reduzida (ICFER).^6^ Define-se como a concentração de hemoglobina < 13,0 g/dl em homens e < 12,0 g/dl em mulheres.^7^ As causas mais comuns de anemia nos pacientes com IC são deficiência de ferro (DF), anemia da doença crônica, dilucional e secundária à insuficiência renal.^8^ A DF ou ferropenia é uma importante comorbidade na IC, estando presente em metade dos pacientes^9 , 10^ não estando restrita apenas aos anêmicos, visto que ocorre em 46% dos pacientes não anêmicos com IC estáveis.^11^

A ferropenia na IC é mais encontrada em pacientes com doença avançada (pior classe funcional e maior valor de peptídeo natriurético cerebral) e em mulheres.^11 , 12^ A presença de ferropenia influencia o prognóstico. Em estudo observacional com 546 pacientes com ICFER, a ferropenia foi apontada como um forte preditor independente de morte ou necessidade de transplante cardíaco, aumentando o risco desses desfechos em aproximadamente 60%.^12^ Em outra coorte constituída de 1506 pacientes europeus com IC crônica, a ferropenia (sem presença de anemia) também foi considerada um preditor de morte.^11^ A alta prevalência e poder prognóstico da ferropenia na IC são justificativas para maior compreensão de sua fisiopatologia, diagnóstico e tratamento.

## Fisiopatologia

### Ferro – Absorção, distribuição e funções no organismo

O ferro é um micronutriente metabolicamente ativo com características bioquímicas únicas. Encontra-se em dois estados oxidativos: ferroso (Fe2+), encontrado no meio intracelular; e férrico (Fe3+), encontrado no meio extracelular.^13^

O consumo médio diário de ferro é de 10–20 mg/dia, mas apenas 10–20% do ferro da dieta é normalmente absorvido utilizando sistemas de transporte específicos, principalmente através dos enterócitos duodenais. O ferro pode ser eliminado por descamação de células mucosas intestinais, menstruação ou outras perdas sanguíneas, porém o corpo não tem um mecanismo fisiologicamente regulado de excreção do ferro, logo a regulação da absorção através do duodeno tem um papel fundamental na homeostase do ferro no organismo.^14^ A maior parte do ferro necessário para eritropoiese (20-25mg) é derivado da reciclagem dos eritrócitos senescentes através da fagocitose dos macrófagos do sistema reticuloendotelial.^13 , 15^

O ferro é distribuído principalmente na hemoglobina das hemácias (65%). Aproximadamente 10% é encontrado em fibras musculares (na mioglobina). O restante é armazenado no fígado, macrófagos do sistema reticuloendotelial e medula óssea.^16^

O ferro tem um papel fundamental no transporte de oxigênio por meio da hemoglobina e armazenamento de oxigênio por parte da mioglobina (células musculares esquelética e cardíaca). Age como componente das enzimas envolvidas na oxidação (fosforilação oxidativa e geração de energia) e das proteínas ferro enxofre (Fe-S) e Heme da cadeia respiratória das mitocôndrias. Também atua na síntese e degradação de proteínas, lipídeos e ácidos ribonucleicos.^13 , 17^

O ferro é potencialmente tóxico porque causa a redução de moléculas de oxigênio dentro da célula, levando à formação de espécies reativas de oxigênio. Logo, o ferro necessita de um neutralizante intracelular na forma de ferritina e intravascular pela ligação à transferrina.^9^

A transferrina age como um reservatório de ferro solúvel. Trata-se de uma glicoproteína transportadora de ferro para células alvo como células eritróides, imunológicas, musculares e hepatócitos. O ferro ligado à transferrina penetra nas células utilizando o receptor de transferrina tipo 1 (TfR1) via endocitose.^16^

O ferro encontra-se armazenado no fígado, medula óssea e baço na forma de ferritina, considerada a principal proteína de reserva do ferro. Em situações de sobrecarga de ferro ou inflamação, ocorre aumento da ferritina tecidual.^13^

A hepcidina é um peptídeo hormonal produzido principalmente pelos hepatócitos, sendo considerada a principal reguladora do metabolismo do ferro.^15^ Sua síntese é regulada pelas mudanças de demanda de ferro no organismo; tem ação direta sobre a ferroportina, um canal transmembrana de ferro. A ferroportina localiza-se na superfície de enterócitos duodenais, responsáveis pela absorção de ferro, assim como nos hepatócitos e macrófagos, responsáveis pelo armazenamento de ferro. Quando ocorre a ligação da hepcidina com a ferroportina, essa é destruída dentro do lisossomo, causando menor liberação de ferro.^13 , 15 , 18^

Em estudo com ratos submetidos à dieta deficiente em ferro durante 12 semanas, comparados aos controles, os animais deficientes em ferro apresentaram coração de peso e tamanho maiores. O exame por microscopia revelou desorganização estrutural dos sarcômeros e mitocôndrias aberrantes no tecido miocárdico.^19^

**Figura 1:**
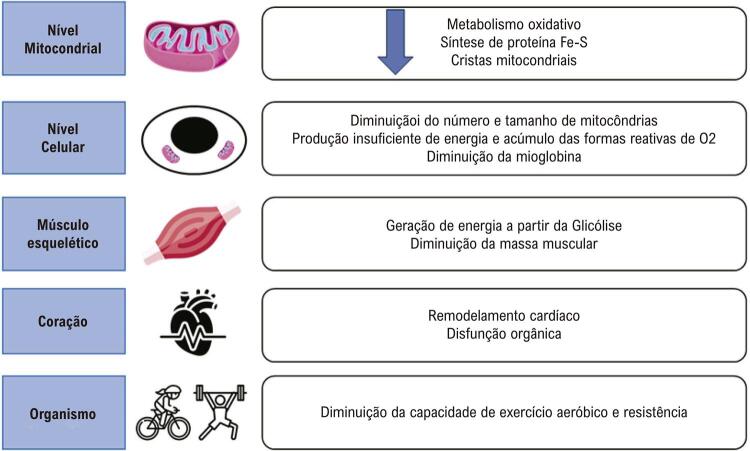
– Efeitos prejudiciais da deficiência de ferro em diferentes níveis de complexidade do organismo (adaptado de Jankowska et al.^13^ e Stugiewicz et al.^20^). Fe-S: ferro-enxofre; O2: oxigênio.

A ferropenia no organismo pode causar efeitos deletérios desde em estruturas mais básicas, como mitocôndrias e células, até níveis mais complexos ( Figura 1 ).^13 , 20^

Estudo com portadores de IC avançada submetidos a transplante cardíaco mostrou depleção de ferro intramiocárdico nesses pacientes em comparação a corações (saudáveis) selecionados para o transplante, sugerindo que a ferropenia intramiocárdica pode ter um papel na patogênese e progressão da IC.^21^

Não apenas a DF causa a IC, como também a IC parece ser capaz de induzir DF, logo a teoria de um ciclo vicioso pode ser formulada.^10^ O surgimento da ferropenia pode ser resultante da baixa captação de ferro devido à desnutrição e sobrecarga de volume; sangramentos associados com uso de antiplaquetários e anticoagulantes, e desordem na utilização e armazenamento do ferro devido ao estado inflamatório da IC.^22 , 23^

Pacientes com estados inflamatórios crônicos como IC, doença renal crônica, câncer e doença inflamatória intestinal apresentam maior risco de desenvolver ferropenia.^9^ Pacientes com IC apresentam aumento na produção de hepcidina no fígado, como já visto, que compromete a absorção de ferro do trato gastrintestinal e a mobilização de ferro proveniente dos estoques de ferro incluindo o sistema reticuloendotelial.^13 , 23 , 24^

### Diagnóstico

A distinção da anemia com ferropenia de anemia da doença crônica é notoriamente difícil. Na ausência de inflamação, níveis séricos de ferritina < 30ng/mL indicam ferropenia.^25^ Em trabalho com pacientes com IC avançada e anemia, foi medido o conteúdo de ferro na biópsia de medula óssea; 73% tinham reservas de ferro reduzidas, sendo que a média de ferritina foi 75 ng/mL nos pacientes com DF e 211 ng/mL nos pacientes sem deficiência. Na IC a ferritina pode estar aumentada ou com nível normal devido ser uma proteína de fase aguda, mesmo em situações como a ferropenia. Logo, o uso de marcadores bioquímicos e pontos de corte convencionais derivados de coortes não-inflamatórias para identificar ferropenia na IC é questionável.^26^

Há dois tipos de ferropenia: absoluta, a qual reflete as reservas de ferro depletadas, com homeostase do ferro e eritropoiese preservadas; e funcional, onde o suprimento de ferro está inadequado para atender a demanda apesar das reservas de ferro normais ou abundantes, devido o ferro estar preso dentro das células do sistema reticuloendotelial e estar indisponível para o metabolismo celular.^13^

Em pacientes com ICFER, convencionou-se ferropenia absoluta como ferritina < 100mg/L, e ferropenia funcional como ferritina 100-299 mg/L e saturação de transferrina (TSAT) <20%.^27 - 29^

### Ferropenia - Um alvo terapêutico

Foram realizados vários ensaios clínicos randomizados (ECRs) para tratamento da DF na ICFER estável ou crônica ( Tabela 1 ). O IRON-HF^30^ foi o primeiro ECR a comparar uso de ferro oral, endovenoso e placebo. Não houve diferença estatística da variação do VO2 de pico entre os grupos. O ensaio foi finalizado antes do previsto devido recrutamento prolongado e problemas de financiamento. Em outro estudo, o IRONOUT-HF, o uso de ferro oral foi comparado com placebo e, novamente, não houve diferença na variação do VO2 de pico.^31^ Esses estudos corroboram o fato de que o ferro por via oral não traz benefício clínico em pacientes com ICFER e DF.

**Tabela 1:** – Ensaios clínicos randomizados com tratamento de deficiência de ferro em pacientes com insuficiência cardíaca

	Toblli et al.^33^	FERRIC-HF^32^	FAIR-HF^34^	IRON-HF^30^	CONFIRM-HF^35^	EFFECT-HF^58^	IRONOUT-HF^31^
n	SHF: 20 Placebo: 20	SHF: 24 Placebo: 11	CF: 304 Placebo: 155	SHF: 10 SF: 7; Placebo: 6	CF: 150 Placebo: 151	CF: 86 Terapia padrão: 86	PF: 111 Placebo: 114
Cegamento	Duplo-cego	Aberto	Duplo-cego	Duplo-cego	Duplo-cego	Aberto	Duplo-cego
Centro(s)	Multicêntrico	Unicêntrico	Multicêntrico	Multicêntrico	Multicêntrico	Multicêntrico	Multicêntrico
Sintomas (CF da NYHA)	II-IV	II-III	II-III	II-IV	II-III	II-III	II-IV
FEVE	≤35%	≤45%	≤40% ou ≤45%	<40%	≤45%	≤45%	≤40%
Definição de DF	Ferritina<100ng/mL e/ou TSAT<20%	Ferritina<100ng/mL ou ferritina 100-299ng/mL + TSAT<20%	Ferritina<100ng/mL ou ferritina 100-299ng/mL + TSAT<20%	Ferritina < 500 μg/L e TSAT<20%	Ferritina<100ng/ml ou ferritina 100-299ng/ml + TSAT<20%	Ferritina<100ng/mL ou ferritina 100-299ng/mL + TSAT<20%	Ferritina<100ng/mL ou ferritina 100-299ng/mL + TSAT<20%
Hb	<12,5 g/dL	<12,5 g/dL (anêmicos), 12,5-14,5 g/dL (não-anemicos)	9-13,5 g/dL	9-12 g/dL	<15 g/dL	<15 g/dL	9-13,5 g/dL
Via do Ferro	Injetável	Injetável	Injetável	Injetável e Oral	Injetável	Injetável	Oral
Tipo de Ferro	SHF	SHF	CF	SHF e SF	CF	CF	PF
Fase de correção (dosagem)	200mg/sem 5 sem	200mg/sem 4 sem	200mg/sem*	SHF 200mg/sem SF 200mg 3xd	500-2000mg semanas 0 e 6	500-2000mg semanas 0 e 6	150mg 2xd 16sem
Fase de manutenção (dosagem)	-	200mg/mês	200mg/mês	-	500mg a cada 12 sem^†^	500mg a cada 12 sem^†^	-
Duração tratamento	5 sem	16 sem	24 sem	5 sem (SHF) 8 sem (SF)	36 sem	12 sem	16 sem
Seguimento	24 sem	18 sem	24 sem	12 sem	52 sem	24 sem	16 sem
Desfecho primário de interesse	Mudança no NT-proBNP e PCR	Mudança no pVO2	Mudança na classe funcional NYHA e no PGA	Mudança no pVo2	Mudança no teste da caminhada de 6 minutos	Mudança no pVo2	Mudança no pVo2
Diferença no desfecho primário	Sim	Não	Sim	Não	Sim	Sim	Não

* Dose calculada de acordo com fórmula de Ganzoni. ^†^ se deficiência de ferro persistir. CF: carboximaltose férrica; DF: deficiência de ferro; FEVE: fração de ejeção do ventrículo esquerdo; Hb: hemoglobina; CF da NYHA: classe funcional da New York Heart Association; PCR: proteína C reativa; PF: polissacarídeo de ferro; PGA: Patient Global Assessment; pVO2: pico do consumo máximo de oxigênio; sem: semana(s); SF: sulfato ferroso; SHF: sacarato de hidróxido férrico; TSAT: saturação de transferrina; NT-proBNP: fragmento N-terminal do peptídeo natriurético tipo B.

Enquanto os primeiros estudos de intervenção com ferro endovenoso utilizaram o sacarato de hidróxido férrico,^32 , 33^ os estudos mais recentes utilizaram a carboximaltose férrica, outra forma de ferro parenteral. Em 2009, foi publicado o FAIR-HF, considerado o maior ECR (n=459) comparando reposição de carboximaltose férrica endovenosa com placebo. Os desfechos primários de interesse foram a classificação funcional da *New York Heart Association* (NYHA) para IC e o *Patient Global Assessment* (PGA) com 24 semanas. O PGA é uma ferramenta onde o paciente dá notas sobre gravidade e evolução da sua doença. No braço carboximaltose férrica, 47% apresentavam classe funcional NYHA I ou II após 24 semanas, comparado com 30% dos que receberam placebo (OR para melhora=2,40; IC95%, 1,55-3,71; p<0,001). O PGA na semana 24 foi melhor no grupo intervenção, 50% relataram estar moderadamente ou muito melhor, comparado com 28% no grupo placebo (OR para melhora=2,51; IC95%, 1,75-3,61; p<0,001). Resultados foram semelhantes em pacientes com anemia e sem anemia.^34^

O estudo CONFIRM-HF foi realizado em nove países da Europa com 301 pacientes, com seguimento mais longo (52 semanas) que o FAIR-HF. De forma semelhante, comparou-se a carboximaltose férrica EV com placebo. O desfecho primário foi a mudança do teste de caminhada de 6 minutos da semana 24 em relação ao basal. Observou-se um aumento de 33 ± 11 metros em favor do grupo que recebeu a carboximaltose, o qual foi mantido até o final do seguimento de 52 semanas. O efeito foi observado tanto nos anêmicos como não-anêmicos, reforçando a ideia de que a ferropenia é um alvo terapêutico independente válido.^35^ Essa diferença excedendo 30 metros nos últimos seis meses do estudo foi robusta e clinicamente significativa, visto que em estudos de intervenção prévios, benefícios de tal magnitude foram apenas vistos com ressincronização cardíaca, relatados em revisão sistemática.^36^ Também foi encontrado menor risco de hospitalização por IC descompensada (HR 0,39; IC95%, 0,19–0,82; p=0,009). Não houve diferença no desfecho de morte cardiovascular (HR 0,96; IC95%, 0,42-2,16; p=0,91).

Em metanálise com cinco ECRs, e total de 851 pacientes, comparando o uso de ferro EV com placebo, não houve diferença na mortalidade cardiovascular (OR 0,80; IC95%, 0,39-1,63; p=0,54) e mortalidade por todas as causas (OR 0,83; IC95% 0,43-1,59; p=0,57). A hospitalização por IC, esta foi menos frequente nos pacientes tratados com ferro endovenoso (OR 0,28; IC95% 0,16-0,50; p<0,0001). Importante ressaltar que 89% dos pacientes incluídos na metanálise receberam ferro parenteral na forma de carboximaltose férrica.^37^

Em outra metanálise^38^ com quatro ECRs e 839 pacientes, comparou-se carboximaltose endovenosa exclusivamente com placebo. Houve redução no desfecho primário de hospitalização de causa cardiovascular e mortalidade cardiovascular (RR 0,59; IC95%, 0,40–0,88; p=0,009) no grupo intervenção. Quando analisada a mortalidade cardiovascular como desfecho isolado, não houve diferença entre os grupos (RR 0,84; IC95%, 0,43-1,66; p=0,620).^38^ A partir dos resultados do CONFIRM-HF e das metanálises, a carboximaltose férrica passou a ser considerada capaz de reduzir as hospitalizações por IC ou causa cardiovascular nos pacientes com fração de ejeção do ventrículo esquerdo (FEVE) reduzida, estáveis e sintomáticos.

A ferropenia passou a ser considerada um alvo terapêutico na ICFER estável, independente da presença de anemia. A diretriz europeia^27^ de IC passou a considerar recomendação IIa a reposição com carboximaltose férrica endovenosa em pacientes NYHA II-III para melhora de sintomas, capacidade de exercício físico e qualidade de vida.^27^ No ano seguinte, a diretriz americana ( *American College of Cardiology* /AHA) de IC utilizou recomendação IIb para o uso de ferro endovenoso na ICFER.^29^ Em 2018 foi publicada a diretriz brasileira de IC, sendo abordada a DF na ICFER, independente da presença de anemia. Foi definida como uma recomendação IIa a administração de ferro endovenoso com intuito de aumento da capacidade de exercício, melhora na qualidade de vida e diminuição de hospitalizações.^8^

**Figura 2:**
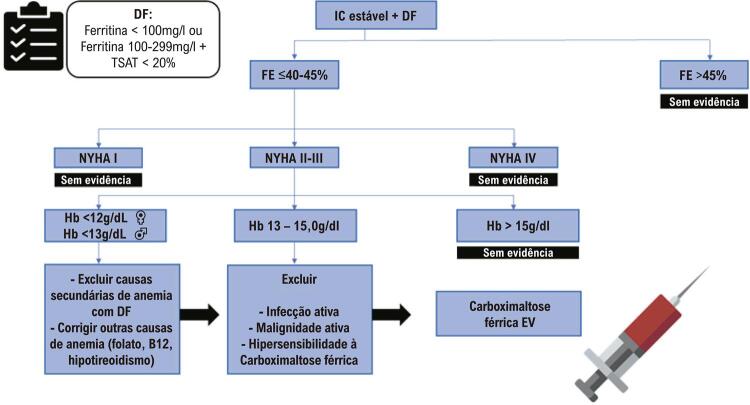
– Algoritmo diagnóstico e terapêutico de pacientes com insuficiência cardíaca e deficiência de ferro (adaptado de Rocha et al.)^10^. DF: deficiência de ferro; EV: endovenoso; FE: fração de ejeção; Hb: hemoglobina; IC: insuficiência cardíaca; NYHA: New York Heart Association; TSAT: saturação de transferrina; DF: deficiência de ferro; EV: endovenosa; HB: hemoglobina; FE: fração de ejeção.

Assim, a identificação dos candidatos à reposição de ferro ( Figura 2 ) é importante e, para isso, é necessário o rastreio de todos os pacientes com IC estáveis e fração de ejeção ≤45%, por meio da medida de ferritina e TSAT.^10 , 27^ A segurança do uso de ferro parenteral é desconhecida em pacientes com IC e hemoglobina >15 g/dL.

O diagnóstico de DF na IC aguda ainda é um desafio. Em estudo observacional com 47 pacientes com IC aguda, o perfil laboratorial do ferro foi obtido no dia da admissão hospitalar e 30º dia. A prevalência de DF foi 83% na admissão, sendo que em 30 dias houve uma queda para 68%. A mediana da ferritina e TSAT foi 93µg/L (IQR: 76–107 μg/L) e 13% (IQR: 6–20%) respectivamente no dia da admissão; com aumento para 159 µg/L (IQR: 134–190 μg/L; p <0,0001) e 17% (IQR: 12–23%; p =0,0176) respectivamente no dia 30, sem qualquer terapia com ferro. Esse estudo demonstra que os marcadores sanguíneos do metabolismo do ferro não são estacionários na IC aguda, mesmo em curto período de observação, tornando questionável o diagnóstico de ferropenia no cenário de descompensação aguda.^39^

Existem outros exames laboratoriais a serem usados na investigação de DF, como o receptor solúvel de transferrina (sTfR) e a hepcidina. No cenário de IC aguda especialmente, o sTfR com valor ≥ 1,59 ng/mL e a hepcidina <14,5 ng/mL parecem ser mais adequados para revelar a DF.^40^ Além disso, foi encontrado que o sTfR tem valor prognóstico na IC, pois seu nível aumentado esteve associado com pior classe funcional de NYHA (p <0,05).^41^

### Ferro no miocárdico

O diagnóstico de DF na IC é relativamente simples de realizar, pois depende apenas de exames laboratoriais (ferritina e TSAT). Em estudo pré-transplante de pacientes com IC avançada, realizaram-se biópsias de miocárdio dos ventrículos para mensurar o ferro miocárdico, investigar sua correlação com marcadores sanguíneos, e compará-los a de doadores (coração saudável). Não foi encontrada correlação entre o ferro intramiocárdico e TSAT, ferritina, ou ferro sérico.^42^ Isso reforça que o metabolismo sistêmico do ferro e do ferro miocárdico são em parte independentes.^43^

### Ressonância Magnética Cardíaca

A Ressonância Magnética Cardíaca (RMC) é uma ferramenta útil para avaliação de pacientes com IC, fornecendo informações a respeito da etiologia e prognóstico.^44^ Anderson et al.^45^ desenvolveram a técnica da sequência T2* (T2-estrela) dentro da RMC, e demonstraram que valores de T2* < 20 ms estão associados à sobrecarga de ferro miocárdico e disfunção ventricular.^45^

Pelo fato da sequência T2* ter sua utilidade estabelecida na sobrecarga de ferro miocárdico, passou-se a questionar se seria capaz de detectar a ferropenia no miocárdio. Em estudo caso-controle de pacientes portadores de IC submetidos à RMC, foi sugerido que um valor de T2* maior estaria relacionado com menor conteúdo de ferro intramiocárdico.^46^ Em um ECR duplo-cego com pacientes portadores de IC sintomáticos (NYHA II e III), fração de ejeção < 50% e ferropenia, os pacientes receberam carboximaltose férrica ou placebo. O desfecho primário foi mudança das sequências T2* e T1 na RMC com sete dias e 30 dias após tratamento. O T2*(ms) foi menor no grupo que recebeu carboximaltose férrica com sete dias [36,6 (34,6–38,7) versus 40 (38–42,1); p=0,025] e 30 dias [36,3 (34,1–38,5) versus 41,1 (38,9–43,4), p=0,003]. Essas mudanças no T2* foram sugestivas de repleção miocárdica após a terapia com carboximaltose.^47^

Até o momento não está estabelecido um ponto de corte de T2* para definição de ferropenia miocárdica, logo a utilidade dessa ferramenta não invasiva na avaliação de pacientes com ferropenia ainda merece mais investigação.

### Tratamento

Na Tabela 2 , encontram-se recomendações de dose da carboximaltose férrica. Depois da correção da ferropenia, considera-se reavaliar os marcadores do ferro (ferritina e TSAT) 1-2x ao ano.^23^

**Tabela 2:** – Dose de carboximaltose férrica endovenosa em pacientes com insuficiência cardíaca e deficiência de ferro10

Peso e Hb	Fase de correção		Fase de manutenção			

	Sem 0	Sem 6	Sem 12	Sem 24	Sem 36	Sem >36
35-70 Kg e Hb <10g/dL	1000 mg	500 mg	500mg se DF persiste	500mg se DF persiste	500mg se DF persiste	Sem evidência
35-70 Kg e Hb ≥10g/dL	1000 mg	0 mg				
> 70 Kg e Hb <10g/dL	1000 mg	1000 mg				
> 70 Kg e Hb ≥10g/dL	1000 mg	500 mg				

*Tabela adaptada de Rocha et al.10 DF: deficiência de ferro; Hb: hemoglobina; IC: insuficiência cardíaca; Sem: semana(s).*

A carboximaltose férrica demonstrou ser custo-efetiva a partir de mudança de classe funcional dos pacientes e redução na taxa de hospitalização.^48^ Comparativamente a outras formulações EV, a carboximaltose férrica é infundida menos vezes, logo o custo total do tratamento pode ser menor,^49^ além de um bom perfil de segurança. Os efeitos indesejáveis dificilmente levam à suspensão da droga. Os efeitos adversos mais comuns (1-10% dos casos) são flushing, tontura, hipertensão arterial, cefaleia, hipofosfatemia e reações locais no sítio de infusão (dor, e descoloração ou irritação da pele).^50^ Pacientes devem ser observados por pelo menos 30 minutos depois da injeção endovenosa para avaliar a ocorrência de efeitos adversos. As contraindicações para o uso da carboximaltose férrica são: hipersensibilidade à carboximaltose ou seus excipientes; hipersensibilidade séria a outro produto parenteral contendo ferro; anemia não atribuída à ferropenia e evidência de sobrecarga de ferro ou distúrbios na utilização do ferro.^23^

### Tratamento da ferropenia na IC aguda

Diferentemente dos outros ensaios já citados em que os participantes eram pacientes estáveis (ambulatoriais), o ECR multicêntrico AFFIRM-AHF, recentemente publicado, incluiu pacientes com FEVE < 50% e DF hospitalizados por IC aguda. Após estabilizados e antes da alta hospitalar os participantes receberam carboximaltose férrica ou placebo por 24 semanas. O desfecho primário foi um composto de hospitalizações totais por IC e morte cardiovascular com 52 semanas, o qual não houve diferença entre os grupos (RR 0,79; IC95%, 0,62-1,01; p=0,059). O desfecho isolado de morte cardiovascular não foi diferente (HR 0,96; IC95% 0,70-1,32; p=0,81), enquanto hospitalizações totais por IC foi menor no braço carboximaltose (RR 0,74; IC95% 0,58-0,94; p=0,013).^51 , 52^ Esta é uma evidência científica atual e relevante, visto que corrobora a indicação de reposição com carboximaltose férrica para pacientes hospitalizados com ICFER e DF com objetivo de diminuir o risco de nova hospitalização por IC.

### Áreas de incerteza

Os critérios para ferropenia utilizados em vários ECRs foram arbitrariamente definidos, sem serem sido validados com a dosagem de ferro no aspirado de medula óssea, considerado o método padrão ouro. Em trabalho^53^ realizado com portadores de IC com FEVE ≤ 45% submetidos à cirurgia de revascularização miocárdica (n=42), foi realizada a dosagem dos marcadores do ferro (ferro sérico, ferritina e TSAT) e aspirado de medula óssea do esterno. A DF foi confirmada na medula óssea em 40% dos pacientes. A partir do diagnóstico de ferropenia da medula óssea, a TSAT ≤ 19,8% apresentou sensibilidade de 94,1% e especificidade de 84% para diagnóstico de ferropenia, enquanto ferro sérico ≤ 13 μmol/L teve sensibilidade 94% e especificidade de 88%. Em contrapartida, a ferritina com valor ≤ 145 ng/mL teve sensibilidade 70,6% e especificidade 60%.^53^ Trata-se de um estudo pequeno, mas levanta a questão se a TSAT e o ferro sérico teriam maior valor para o diagnóstico de ferropenia em vez da ferritina.

A maioria dos pacientes incluídos em ECRs (FAIR, CONFIRM e EFFECT) apresentavam DF absoluta (80-90%), enquanto a DF funcional foi pouco representada.^10^ Em estudo transversal realizado com pacientes com ICFER, os pacientes foram classificados dentro de categorias: transporte de ferro reduzido (TSAT < 20%); DF absoluta (ferritina < 100 μg/L); e status do ferro normal. Os pacientes com transporte de ferro reduzido isoladamente apresentaram níveis maiores de fragmento N-terminal do peptídeo natriurético tipo B (NT-proBNP) e pior qualidade de vida [OR 1,7 (1,2–2,5); p=0,005] quando comparados àqueles com status de ferro normal [OR 2,1 (1,5–2,9) p<0,001], e não houve diferença do NT-proBNP e qualidade de vida quando comparados os grupos com DF absoluta e status do ferro normal.^54^ Os achados desse estudo tornam relevante a discussão sobre a importância dos pacientes com TSAT <20% ou DF funcional terem maior representatividade nos ECRs.

Até a presente data, os ECRs já realizados não foram desenhados com poder suficiente para avaliar o benefício do ferro endovenoso em reduzir mortalidade em pacientes com ICFER estáveis. Está em andamento o ECR duplo-cego placebo controlado, FAIR-HF 2,^55^ o qual pretende recrutar pacientes com ICFER e ferropenia de modo a avaliar se a carboximaltose férrica é capaz de reduzir o desfecho primário combinado de hospitalização por IC e morte cardiovascular.

A maior parte da evidência científica de ferropenia e IC origina-se de estudos de pacientes com fração de ejeção reduzida. Existe uma lacuna de conhecimento a respeito de estudos de intervenção na ferropenia e IC com fração de ejeção preservada (ICFEP). Em revisão sistemática e metanálise com 1877 pacientes com ICFEP, a prevalência de ferropenia foi de 59%. Os portadores de ferropenia apresentaram pior classe funcional, capacidade de exercício e qualidade de vida quando comparados aos sem ferropenia. Não houve diferença quanto ao risco de morte ou de hospitalização.^56^ Outro ECR, o FAIR-HFpEF,^57^ está sendo conduzido para avaliar a eficácia e a segurança da reposição de carboximaltose férrica nos pacientes com ICFEP e ferropenia.

## Conclusões

A ferropenia é uma comorbidade muito comum nos portadores de ICFER e passou a ser considerada um alvo terapêutico. A carboximaltose férrica endovenosa melhora os sintomas, capacidade de exercício físico e qualidade de vida nos pacientes com ICFER estáveis, sintomáticos e com FEVE ≤45%, tanto nos anêmicos como não anêmicos. Também há evidência de redução do risco de hospitalização por IC. Em contrapartida, a formulação de ferro oral não traz benefício clínico em pacientes com ICFER e DF. Até o momento não há evidência científica que sustente a indicação de carboximaltose férrica nos pacientes com ICFEP.
