# Supplementary material for: Deficiência de Ferro na Insuficiência Cardíaca com Fração de Ejeção Reduzida: Fisiopatologia, Diagnóstico e Tratamento
Source: Arq Bras Cardiol. 2022 Mar 10;118(3):646–54. [Article in Portuguese] doi: 10.36660/abc.20201257 (PMC8959039; doi:10.36660/abc.20201257)
Supplement: Translation

# Translation: Iron Deficiency in Heart Failure with Reduced Ejection Fraction: Pathophysiology, Diagnosis and Treatment

**Authors:** Guilherme Augusto Reissig Pereira, Luís Beck-da-Silva

**Affiliations:** 1 Brasil Hospital São Lucas da Pontifícia Universidade Católica do Rio Grande do Sul, Porto Alegre, RS – Brasil; 2 Brasil Programa de Pós-Graduação em Cardiologia – Universidade Federal do Rio Grande do Sul, Porto Alegre, RS – Brasil; 3 Brasil Hospital de Clínicas de Porto Alegre, Porto Alegre, RS – Brasil

**Keywords:** Iron, Iron Deficiency, Heart Failure, Systolic

## Abstract

Iron deficiency (ID) is an important comorbidity in heart failure with reduced ejection (HFrEF) and is highly prevalent in both anemic and non-anemic patients. In HFrEF, iron deficiency should be investigated by measurements of transferrin saturation and ferritin. There are two types of ID: absolute deficiency, with depletion of iron stores; and functional ID, where iron supply is not sufficient despite normal stores. ID is associated with worse functional class and higher risk of death in patients with HFrEF, and scientific evidence has indicated improvement of symptoms and quality of life of these patients with treatment with parenteral iron in the form of ferric carboxymaltose. Iron plays vital roles such as oxygen transportation (hemoglobin) and storage (myoblogin), and is crucial for adequate functioning of mitochondria, which are composed of iron-based proteins and the place of energy generation by oxidative metabolism at the electron transport chain. An insufficient generation and abnormal uptake of iron by skeletal and cardiac muscle cells contribute to the pathophysiology of HF. The present review aims to increase the knowledge of the pathophysiology of ID in HFrEF, and to address available tools for its diagnosis and current scientific evidence on iron replacement therapy.

## Clinical issue

Heart failure (HF) is a global health problem that affects 26 million people in the world.^1^ In Brazil, the number of patients with HF was approximately 2,846,000 in 2015, with increasing prevalence with age.^2^

In a Brazilian registry of patients hospitalized for HF in different parts of the country, in-hospital mortality was 12.6%.^3^ In addition to the high in-hospital mortality, it is estimated that nearly 50% of patients diagnosed with HF will die within five years.^4 , 5^ Also, HF has a strong economic impact, leading to a cost of 22.1 billion Brazilian reals in 2015.^2^

Anemia is a common problem in HF with reduced ejection fraction (HFrEF).^6^ It is defined as hemoglobin (Hb) levels <13.0 g/dL in men and <12.0 g/dL in women.^7^ The most common causes of anemia are iron deficiency (ID), chronic diseases, dilutional anemia and renal failure.^8^ ID is a common comorbidity in HF, affecting nearly half of HF patients;^9 , 10^ it is not restricted to anemic patients, since 46% of patients without anemia with stable HF has ID.^11^

ID in HF is more commonly seen in patients with more advanced disease (worse functional class and higher brain natriuretic peptide levels) and in female patients.^11 , 12^ The presence of ID affects the prognosis. In an observational study with 546 patients with HFrEF, ID was a strong independent predictor of death or need for heart transplantation, increasing the risk for these outcomes by nearly 60%.^12^ In another cohort composed of 1,506 European patients with chronic HF, ID (without anemia) was also considered a predictor of death.^11^ The high prevalence and the strong prognostic power of ID in HF warrant a better understanding of its pathophysiology, diagnosis and treatment.

## Pathophysiology

### Iron – Absorption, distribution and functions in the body

Iron is a metabolically active micronutrient with unique biochemical features. It has two oxidation states – ferrous (+2) and ferric (+3), found inside and outside cells, respectively.^13^

Mean daily intake of iron is 10-20mg/day, although only 10-20% of dietary iron is actually absorbed through specific transportation systems, especially duodenal enterocytes. Iron can be eliminated from the body by desquamation of intestinal mucosal cells, menstruation, or other blood losses. However, because of the lack of a physiologically regulated excretion system for iron in the body, the regulation of its absorption through the duodenum plays a crucial role in iron homeostasis in the body.^14^ Most iron required for erythropoiesis (20-25 mg) is derived from recycling of senescent erythrocytes from macrophage phagocytosis in the reticuloendothelial system.^13 , 15^

Regarding iron distribution in human body, approximately 65% of the mineral is found in hemoglobin of erythrocytes, and nearly 10% is found in myoglobin of muscle fibers. The remaining is stored in the liver, macrophages of the reticuloendothelial system, and bone marrow.^16^

Iron plays a fundamental role in oxygen transport by hemoglobin and in oxygen storage in the myoglobin of skeletal and cardiac muscle cells. Iron acts as a component of enzymes involved in oxidation (oxidative phosphorylation) and of iron-sulfur and heme proteins in the respiratory chain of mitochondria. Iron participates in the synthesis and degradation of proteins, lipids and ribonucleic acids.^13 , 17^

Iron is a potentially toxic metal as it causes the reduction of oxygen molecules in the cells, resulting in the formation of oxygen reactive species. Thus, iron requires an intracellular and an extracellular neutralizer, in the form of ferritin and transferrin, respectively.^9^

Transferrin is a glycoprotein that acts as a storage depot and mediates the transport of soluble iron. Transferrin receptor 1 (TfR1) mediates the uptake of transferrin-bound iron by receptor-mediated endocytosis.^16^

Iron is stored in the liver, bone marrow and spleen in the form of ferritin, which is the main storage protein of iron. Concentrations of tissue ferritin increase in situations of iron overload or inflammation.^13^

Hepcidin is a hormone peptide produced mainly by hepatocytes and is considered the main regulator of iron metabolism.^15^ Its synthesis is regulated by changes in iron requirements in the body. Hepcidin directly acts on ferroportin, a transmembrane protein that transports iron. Ferroportin is located on the surface of duodenal enterocytes, responsible for iron absorption, and on hepatocytes and macrophages, responsible for iron storage. When hepcidin binds ferroportin, the transporter is degraded in lysosomes, resulting in reduced iron release.^13 , 15 , 18^

In an experimental study, rats receiving a diet deficient in iron for 12 weeks exhibited increased heart weight and size compared with the control group. Analysis by microscopy revealed abnormal sarcomere structure and mitochondrial ultrastructural aberrations in myocardial tissue.^19^

Iron depletion may have deleterious effects in the body, involving since basic structures, such as mitochondria and cells, until more complex ones ( Figure 1 ).^13 , 20^

**Figure 1:** – Harmful effects of iron deficiency at different levels of organism complexity (adapted from Jankowska et al.^13^and Stugiewicz et al.^20^). Fe-S: iron-sulfur; O2: oxygen.

A study on patients with advanced HF undergoing heart transplantation demonstrated depletion of myocardial iron in these patients as compared with healthy controls, suggesting that myocardial iron depletion may play a role in the pathogenesis and progression of HF.^21^ ID causes HF, and HF itself seems to induce ID, suggesting the theory of a vicious cycle.^10^ The development of ID may be resultant of reduced iron uptake due to malnutrition and volume overload, hemorrhage associated with antiplatelet and anticoagulants, and disturbances in iron utilization and storage caused by inflammation in HF.^22 , 23^

Patients with chronic inflammatory conditions such as HF, chronic renal disease, cancer, and inflammatory bowel disease are at higher risk of developing ID.^9^ in HF patients, hepcidin production in the liver is increased, affecting iron absorption in the gastrointestinal tract and iron mobilization from iron stores, including the reticuloendothelial system.^13 , 23 , 24^

### Diagnosis

The distinction between ID anemia and anemia of chronic disease is difficult. In the absence of inflammation, serum levels of ferritin < 30 ng/mL are indicative of ID.^25^ In a study on patients with advanced anemia, bone marrow aspiration was performed, and ID was confirmed in 73% of patients. Mean ferritin was 75 ng/mL in iron deficient patients, and 211 ng/mL in non-iron deficient patients. As ferritin is an acute-phase protein, their levels may be either normal of increased in HF, even in situations of ID. Thus, the use of conventional biomarkers and conventional cut-off points obtained from patients with non-inflammatory conditions to identify ID in HF is questionable.^26^

There are two types of ID: absolute deficiency, which reflects depletion of iron stores, with preserved iron homeostasis and erythropoiesis; and functional ID, where iron supply is not sufficient to meet the requirements despite normal or even excess reserve, because iron is trapped inside cells of the reticuloendothelial system and is not available for cellular metabolism.^13^

In patients with HFrEF, absolute ID was defined as ferritin < 100mg/L, and functional ID as ferritin of 100-299 mg/L and transferrin saturation (TSAT) < 20%.^27 - 29^

### Iron deficiency – a therapeutic target

Several randomized clinical trials (RCTs) of treatment of ID in stable and chronic HFrEF have been performed ( Table 1 ). The IRON-HF^30^ was the first RCT to compare the use of oral iron, intravenous iron and placebo. No statistically significant difference was found in changes of peak VO2 between the groups. Due to prolonged recruitment and financing issues, the trial was stopped before planned. In another study, the IRONOUT-HF, therapy with oral iron was compared with placebo and, again, no difference in peak VO2 was observed between the groups.^31^ These studies corroborate the fact that oral iron supplementation has no clinical benefit in patients with HFrEF and ID.

**Table 1:** – Randomized clinical trials on treatment of iron deficiency in patients with heart failure

	Toblli et al.^33^	FERRIC-HF^32^	FAIR-HF^34^	IRON-HF^30^	CONFIRM-HF^35^	EFFECT-HF^58^	IRONOUT-HF^31^
n	FHS: 20 Placebo: 20	FHS: 24 Placebo: 11	FC: 304 Placebo: 155	FHS: 10 SF: 7; Placebo: 6	FC: 150 Placebo: 151	FC: 86 Standard therapy: 86	FP: 111 Placebo: 114
Bliding	Double-blind	Open	Double-blind	Double-blind	Double-blind	Open	Double-blind
Center (s)	Multicentric	Single-center	Multicentric	Multicentric	Multicentric	Multicentric	Multicentric
Symptoms (NYHA functional class)	II-IV	II-III	II-III	II-IV	II-III	II-III	II-IV
LVEF	≤35%	≤45%	≤40% or ≤45%	<40%	≤45%	≤45%	≤40%
ID definition	Ferritin<100ng/mL and/or TSAT<20%	Ferritin<100ng/mL or ferritin 100-299ng/mL + TSAT<20%	Ferritin<100ng/mL or ferritin 100-299ng/mL + TSAT<20%	Ferritin < 500 μg/L and TSAT<20%	Ferritin<100ng/mL or ferritin 100-299ng/mL + TSAT<20%	Ferritin<100ng/mL or ferritin 100-299ng/mL + TSAT<20%	Ferritin<100ng/mL or ferritin 100-299ng/mL + TSAT<20%
Hb	<12.5 g/dL	<12.5 g/dL (anemic), 12.5-14.5 g/dL(non-anemic)	9-13.5 g/dL	9-12 g/dL	<15 g/dL	<15 g/dL	9-13.5 g/dl
Iron pathway	Injectable	Injectable	Injectable	Injectable and oral	Injectable	Injectable	Oral
Type of iron	FHS	FHS	FC	FHS and FS	FC	FC	PF
Correction phase (dosage)	200mg/week 5 weeks	200mg/week 4 weeks	200mg/week*	SHF 200mg/week SF 200mg 3xd	500-2000mg week 0 and 6	500-2000mg week 0 and 6	150mg 2xd 16sem
Maintenance phase (dosage)	-	200mg/month	200mg/month	-	500mg every 12 weeks^†^	500mg every 12 weeks^†^	-
Treatment duration	5 weeks	16 weeks	24 weeks	5 weeks (SHF) 8 weeks (FS)	36 weeks	12 weeks	16 weeks
Follow-up	24 weeks	18 weeks	24 weeks	12 weeks	52 weeks	24 weeks	16 weeks
Primary outcome of interest	Change in NT-proBNP and CRP	Change in pVO2	Change in NYHA FC and PGA	Change in pVO2	Change in the six-minute walk test distance	Change in pVO2	Change in pVO2
Difference in primary outcome	Yes	No	Yes	No	Yes	Yes	N

* Calculated using the Ganzoni equation.^†^if iron deficiency persists. FC: ferric carboxymaltose; ID: iron deficiency; LVEF: left ventricular ejection fraction; Hb: hemoglobin; CRP: C-reactive protein; IP: iron polysaccharide; PGA: Patient Global Assessment; pVO2: peak oxygen consumption; FS: ferrous sulfate; FHS: ferric hydroxide saccharate; TSAT: transferrin saturation; NT-proBNP: N-terminal B-type natriuretic peptide fragment; NYHA: New York Heart Association.

While the first interventional studies with intravenous iron used ferric hydroxide saccharate complex,^32 , 33^ more recent trials used ferric carboxymaltose, another form of parenteral iron. In 2009, the FAIR-HF, considered the largest RCT comparing intravenous administration of ferric carboxymaltose with placebo. Primary outcomes of interest were New York Heart Association (NYHA) functional class and Patient Global Assessment (PGA) at 24 weeks. PGA is a rating scale on which patients rate disease severity and progression. In the ferric carboxymaltose arm, 47% showed improvement in NYHA functional class (to I or II) at 24 weeks, compared with 30% of those who received placebo (OR =2.40; 95%CI 1.55-3.71; p<0.001). PGA at week 24 was better in the interventional group, where 50% of patients reported a moderate or marked improvement, compared with 28% in the placebo group (OR for improvement 2.51; 95% CI 1.75-3.61; p<0.001). Results were similar in patients with and without anemia.^34^

The CONFIRM-HF study was performed in nine countries in Europe, including 301 patients, with a longer follow-up period (52 weeks) compared with the FAIR-HF. Both studies compared the use of intravenous ferric carboxymaltose with placebo. Primary outcome was improvement in six-minute walk test at 24 weeks compared with baseline. There was an increase in distance walked by 33 ± 11 meters in the group who received carboxymaltose, until the end of the follow-up period at 52 weeks. The effect was observed in both anemic and non-anemic patients, reinforcing the idea that ID is a valid independent therapeutic target.^35^ This difference of more than 30 meters in the last six months of study was robust and clinically significant, especially considered that in previous interventional studies, benefits of this magnitude have been reported with cardiac resynchronization by a systematic review.^36^ Lower risk of hospitalization was also found in decompensated HF (HR 0.39; 95%CI 0.19–0.82; p=0.009). No difference was found in cardiovascular mortality outcome (HR 0.96; 95%CI, 0.42-2.16; p=0.91).

In a meta-analysis with five RCTs and 851 patients comparing intravenous iron with placebo, no difference found in cardiovascular mortality (OR 0.80; 95%CI 039-1.63; p=0.54) or all-cause mortality (OR 0.83; 95%CI 0.43-1.59; p=0.57). Hospitalization for HF was less frequent in patients treated with intravenous iron (OR 0.28; 95%CI 0.16-0.50; p<0.0001). It is worth pointing out that 89% of patients included in this meta-analysis received parenteral iron in the form of ferric carboxymaltose.^37^

Another meta-analysis with four RCTs and 839 patients compared administration of intravenous carboxymaltose with placebo; there was a reduction in cardiovascular hospitalizations and cardiovascular mortality (RR 0.59; 95%CI 0.40–0.88; p=0.009) in the intervention group. When cardiovascular mortality was analyzed alone, no difference was found between the groups (RR 0.84; 95%CI, 0.43-1.66; p=0.620).^38^ Based on the results of the CONFIRM-HF study and other meta-analyses, ferric carboxymaltose has been considered effective in reducing HF or cardiovascular hospitalizations in stable, symptomatic, patients with reduced left ventricular ejection fraction (LVEF).

Then, ID has become a therapeutic target in stable HFrEF, independent of the presence of anemia. The European guidelines on HF^27^ have considered intravenous administration of ferric carboxymaltose a IIa recommendation for improvement of symptoms, exercise capacity, and quality of life in NYHA class II/III patients.^27^ In the next year, the American (American College of Cardiology/AHA) guidelines on HF gave a class II b recommendation for intravenous iron in HFrEF.^29^ In 2018, the Brazilian guidelines on HF were published, which addressed ID in HFrEF, independent of the presence of anemia. Intravenous administration of iron was given a IIa recommendation to improve exercise capacity and quality of life and reduce hospitalizations.^8^

Therefore, it is important to identify candidates for iron replacement therapy ( Figure 2 ), by screening of all patients with stable HF and ejection fraction ≤45% by measurement of serum ferritin and TSAT.^10 , 27^ The safety of parenteral iron is still unknown in HF patients with hemoglobin > 15g/dL.

**Figure 2:** – Diagnostic and therapeutic algorithm for patients with heart failure and iron deficiency (adapted from Rocha et al.)10. ID: iron deficiency; NYHA: New York Heart Association; EF: ejection fraction: Hb: hemoblogin; B12: vitamina B12; IV: intravenous; TSAT: transferrin saturation.

The diagnosis of ID in acute HF is still a challenge. In an observational study with 47 patients with acute HF, iron profile was measured at admission and on day 30. The prevalence of ID was 83% at admission, with a decrease to 68% on day 30. Median ferritin and TSAT were 93µg/L (IQR: 76–107 μg/L) and 13% (IQR: 6–20%), respectively, on admission, and 159 µg/L (IQR: 134–190 μg/L; p <0.0001 compared with admission) and 17% (IQR: 12–23%; p =0.0176) respectively on the 30th day, without iron replacement therapy. This study demonstrates that biomarkers of iron metabolism are not steady in acute HF, even in a short period of observation, making the diagnosis of ID in acutely decompensated HF questionable.^39^

Other laboratory tests may be used in the investigation of ID, such as soluble transferrin receptor (sTfR) and hepcidin. In the scenario of acute IC, a sTfR ≥1.59ng/mL and a hepcidin < 14.5ng/mL seem adequate to detect ID.^40^ In addition, sTfR was found to have a prognostic value in HF, as increased sTfR levels were associated with worse NYHA functional class (p<0.05).^41^

### Myocardial iron

The diagnosis of ID in HF is relatively easy to be made, as it depends on laboratory tests (ferritin and TSAT) only. In a study with pretransplant patients with advanced HF, ventricular myocardial biopsies were performed to measure myocardial iron in the explanted failing hearts, compared to non-failing hearts, and to assess the correlation of myocardial iron with serum markers. No correlation was found of myocardial iron with TSAT, ferritin, or serum iron,^42^ reinforcing that the metabolism of systemic iron and myocardial iron are partly independent.^43^

### Cardiac magnetic resonance

Cardiac magnetic resonance (CMR) is a useful tool in the assessment of patients with HF, that provides information regarding its etiology and prognosis.^44^ Anderson et al.^45^ developed the cardiovascular T2-star (T2*) magnetic resonance technique, and demonstrated that a myocardial T2* of <20 ms was associated with myocardial iron overload and ventricular dysfunction.^45^

As myocardial T2* was shown to be useful in the evaluation of myocardial iron overload, studies have been made to test its utility in detecting myocardial ID also. In a case-control study with HF patients undergoing CMR, a higher T2* seemed to be related with lower myocardial iron content.^46^ In a double-blind RCT with symptomatic HF patients (NYHA II and III), ejection fraction < 50% and ID, patients received either ferric carboxymaltose or placebo. Primary outcome was changes in magnetic resonance T2* and T1 at seven and 30 days of treatment. T2* (ms) was significantly lower in the ferric carboxymaltose arm on day seven (36.6 [34.6–38.7] versus 40 [38–42.1], p=0.025) and day 30 (36.3 [34.1–38.5] versus 41.1 [38.9–43.4], p=0.003). These changes in T2* were suggestive of myocardial iron repletion with ferric carboxymaltose administration.^47^

So far, the cut-off point of T2* for detecting myocardial ID has not been established, and hence the usefulness of this non-invasive tool in the assessment of patients with ID still requires further investigation.

### Treatment

Recommended therapeutic dosages of ferric carboxymaltose are described in Table 2 . After correction of ID, reevaluation of serum iron markers (ferritin and TSAT) once-twice a year.^23^

**Table 2:** – Dose of intravenous ferric carboxymaltose in patients with heart failure and iron deficiency10

Weight and Hb	Correction phase		Maintenance phase			

	Week 0	Week 6	Week 12	Week 24	Week 36	Week >36
35-70 Kg and Hb <10g/dL	1000 mg	500 mg	500mg if ID persists	500mg if ID persists	500mg if ID persists	No evidence
35-70 Kg and Hb ≥10g/dL	1000 mg	0 mg				
> 70 Kg and Hb <10g/dL	1000 mg	1000 mg				
> 70 Kg and Hb ≥10g/dL	1000 mg	500 mg				

*Table adapted from Rocha et al.^10^ID: iron deficiency; Hb: hemoglobin; HF: heart failure.*

Ferric carboxymaltose has been shown to be cost effective by changes in functional class and reduction in hospitalization rates.^48^ Since the number of infusions of ferric carboxymaltose is relatively lower, as compared with other intravenous formulations, the total cost of treatment may be lower,^49^ in addition to a good safety profile. The therapy is rarely discontinued due to undesirable effects. The most common adverse effects (1-10% of the cases) are flushing, seizure, arterial hypertension, headache, hypophosphatemia, and local reaction on the site of infusion (skin discoloration, pain, irritation).^50^ Patients should be monitored for at least 30 minutes after intravenous injection for the occurrence of adverse effects. Contraindications to the use of ferric carboxymaltose are: hypersensitivity to carboxymaltose and its excipients; severe hypersensitivity to other parenteral formulations containing iron; non-iron-deficiency anemia; and evidence of iron overload or disturbances in iron utilization.^23^

### Treatment of ID in acute HF

Unlike other trials above mentioned, that included stable outpatients, the recently published multicentric RCT AFFIRM-AHF included patients with LVEF < 50% and ID hospitalized for acute HF. After stabilization and before hospital discharge, participants received ferric carboxymaltose or placebo for 24 weeks. The primary composite outcome was total admissions for HF and cardiovascular death within 52 weeks, which was not different between the groups (RR 0.79; 95%CI, 0.62-1.01; p=0.059). The outcome of cardiovascular death alone was not different (HR 0.96; 95%CI, 0.70-1.32; p=0.81), whereas total admissions for HF was lower in the carboxymaltose (RR 0.74; 95%CI, 0.58-0.94; p=0.013).^51 , 52^ This is a relevant, up-to-date scientific evidence, as it corroborates the indication of ferric carboxymaltose supplementation for patients hospitalized for HFrEF and ID, aiming at reducing the risk for readmissions for HF.

### Areas of uncertainty

The criteria to define ID adopted in several RCTs have been arbitrarily established, without validation with iron staining on bone marrow aspirate smears, which is considered the gold-standard method. Grote Beverborg et al.^53^ conducted a study with HF patients with LVEF ≤ 45% undergoing myocardial revascularization surgery (n=42) and performed measurements of iron-related markers (serum iron, ferritin, TSAT) and bone marrow aspiration with iron staining. Bone marrow ID was found in 40% of the HF patients. Based on the diagnosis of ID confirmed by bone marrow aspiration, TSAT ≤ 19,8% had a sensitivity of 94.1% and a specificity of 84%, and serum iron ≤13 µmol/L had a sensitivity of 94% and specificity of 88%. On the other hand, ferritin ≤145 ng/mL had a sensitivity of 70.6% and specificity of 60%.^53^ Although this was a small study, it raised the question on whether TSAT and serum iron would be more important for the diagnosis of ID than ferritin.

Most patients included in RCTs (FAIR, CONFIRM and EFFECT) had absolute ID (80=90%), whereas functional ID was poorly represented.^10^ In a cross-sectional study, patients with HFrEF were categorized into the following: impaired iron transport (TSAT < 20%); absolute ID (ferritin < 100 μg/L); and normal iron status. Patients with isolated impaired iron transport had higher N-terminal pro b-type natriuretic peptide (NT-proBNP) levels (OR 2.1 [1.5–2.9] p<0.001) and worse quality of life (OR 1.7 [1.2–2.5]; p=0,005) as compared with patients with normal iron status, and no difference in NT-proBNP levels compared with patients with absolute ID and normal iron status.^54^ These findings highlight the importance of including patients with TSAT<20% or functional ID in RCTs.

The currently available RCTs do not have enough power to evaluate the benefit of intravenous iron in reducing mortality in patients with stable HFrEF. The ongoing double-blind, placebo-controlled study FAIR-HF 2,^55^ aims to evaluate whether ferric carboxymaltose can reduce the primary composite endpoint of hospitalization for HF and cardiovascular death in patients with HFrEF and ID.

Most of the evidence available to date is based on studies with patients with reduced ejection fraction. There is a gap in knowledge for patients with HF and preserved ejection fraction (HFpEF). In a systematic review and meta-analysis of 1,877 with HFpEF, the prevalence of ID was 59%. Patients with ID had worse functional class, exercise capacity and quality of life compared with those without ID. No difference was found regarding risk of death or hospitalization.^56^ Another RCT, the FAIR-HFpEF,^57^ currently in progress, aims to evaluate the efficacy and safety of ferric carboxymaltose administration in patients with HFpEF and ID.

## Conclusions

ID is a very common comorbidity in patients with HFpEF that has become a therapeutic target. Intravenous ferric carboxymaltose improves symptoms, exercise capacity and quality of life in symptomatic patients with stable HFrEF and LVEF ≤45%, in both anemic and non-anemic patients. There is also evidence of a reduction in the risk of HF. On the other hand, oral iron formulations have no clinical benefits in patients with HFrEF and ID. So far, there is no clinical evidence supporting ferric carboxymaltose administration in patients with HFrEF.
